# Stick to Principle

**Published:** 1878-03-20

**Authors:** 


### STICK TO PRINCIPLE.

A medical friend, Dr. W., of Virginia, in a recent communication, after a very high compliment to the practical features of "The Record," propounds a query relative to an issue which exists in his Medical Society. We thank our friend for his high opinion of our journal, but are not prepared to take sides in the difficulty to which he alludes. There is, however, one rule, which in our own experience, we have endeavored rigidly to adhere to, and that is to find out the principle underlying the matter in controversy, and to openly and boldly espouse that which we believe to be right, no matter whom it offends or whose toes are trod upon. We do not hunt up chances to do this or make it a rule to go out of our own section for this purpose. Yet we do not shirk the truth to "*keep out of difficuliies.*" That which is right and just should ever be adhered to regardless of mere expediency or policy, and we hesitate not to say to our friend that Davy Crocket's motto was a good one—"Be sure you are right, and then go ahead." We regard decision and firmness as an important element in human character, and confess to little sympathy with the merely neutral or negative man, or the man who tries to please all parties. Such an one does little for himself or others. He has no influence, and like the man who falls between two stools, he is more likely to incur ridicule than sympathy. "The double minded man is unstable in all his ways" says the good Book. P.

